# Revisiting the factors influencing the magnetic resonance contrast of Gd_2_O_3_ nanoparticles†

**DOI:** 10.1039/d1na00612f

**Published:** 2021-10-25

**Authors:** Yanyue Liu, Yingfan Dai, Haifeng Li, Dida Duosiken, Na Tang, Kang Sun, Ke Tao

**Affiliations:** State Key Lab of Metal Matrix Composites, School of Materials Science and Engineering, Shanghai Jiao Tong University Shanghai 200240 P. R. China ktao@sjtu.edu.cn

## Abstract

Gadolinium oxide nanoparticles (GONs) have the potential to be one of the best candidates for the contrast agents of magnetic resonance imaging. Even though the influence of parameters on the relaxation has been substantially demonstrated, the variation of the *r*_1_ of GONs with a similar structure and surface chemistry implied our limited understanding. We herein synthesized GONs with adjustable size, shape, and crystallinity, modified them with a series of molecules with different acidities, and recorded their *r*_1_ values and imaging contrast. Our results showed that the isoelectric point could be regarded as an indicator of the relaxation covering the influence of both surface modification and size, which highlighted the impact of protons dissociated from the contrast agents. We further showed that the nanoparticles with lower crystallinity possess higher relaxivity, and this phenomenon manifested significantly under a low field. Our work clarified that the longitudinal relaxivity of Gd_2_O_3_ nanoparticles is sensitively dependent on the numbers of H^+^ generated from the surface and in the environment, which may shed light on developing high-performance nanoparticulate *T*_1_ contrast agents.

## Abstract 2

Magnetic resonance contrast of gadolinium oxide nanoparticles depends on the isoelectric point.

## Graphical abstract

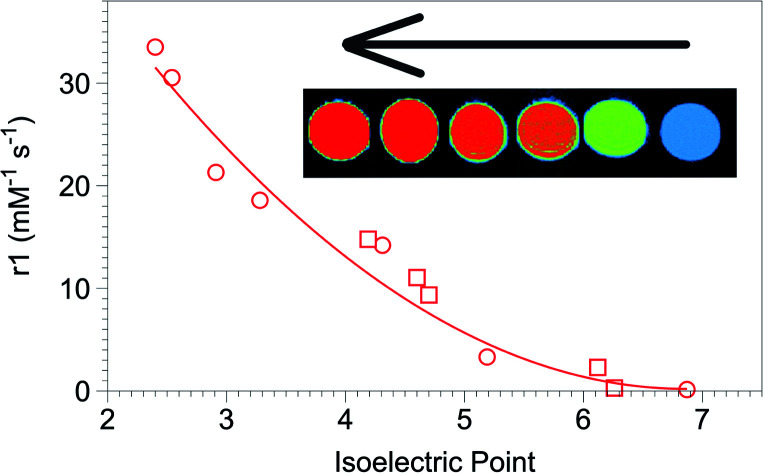

## Introduction

Magnetic resonance imaging (MRI) is a crucial technique for noninvasive diagnosis of diseases.^1,2^ To fulfill the clinical demands of imaging sensitivity, over 40% MRI examinations are conducted with the help of contrast agents.^3–5^ The most important contrast agents that have been approved are gadolinium (Gd) chelates. They inherit the advantages of Gd^3+^ such as a large number of unpaired electrons, high magnetic moment, and the capability of effectively decreasing the longitudinal relaxation time of hydrated protons.^6–11^ However, fast clearance away from sites of interest requires repeated doses within a short time frame, which may increase the risk of toxicity such as eliciting nephrogenic systemic fibrosis.^12–15^ Compared with small molecules, gadolinium oxide nanoparticles (GONs) could be detained at target positions. Meanwhile, they possess a high density of paramagnetic ions, and exhibit a promising enhancement of the longitudinal relaxivity (*r*_1_).^3,16–18^ Therefore, potentiating the *r*_1_ value of GONs to maximize their advantages has attracted intensive attention in recent years.

The influence of size,^17,19,20^ shape,^16,21^ and surface modification^22,23^ of GONs on *T*_1_ relaxation has been highly studied. In general, the relaxivity can be described by Solomon–Bloembergen–Morgan (SBM) theory, in which the key parameters are the hydration number (*q*), proton residence time (*τ*_m_), and rotational correlation time (*τ*_R_). *T*_1_ relaxivity is positively correlated with *q*, 1/*τ*_m_, and *τ*_R_. Decreasing the size or enlarging the surface-to-volume ratio of Gd_2_O_3_ core elevates the relaxivity. Since the direct chemical exchange between GONs and water molecules is the largest contributor to proton relaxation, decreasing the Gd_2_O_3_ core size enhances *T*_1_-weighted MRI by increasing the density of Gd^3+^ ions on the surface, or the parameter *q*.^17,24^ Another pivotal factor is the surface modification, which influences the tumbling time (*τ*_R_), that depends on the viscosity of medium. Assuming that the viscosity is constant for the solvent containing nanoparticles, the viscosity would be elevated in the modified range close to the surface, which increases the tumbling time.^25^ Therefore, the increased hydrodynamic size promotes the relaxivity. This explained that Gd_2_O_3_ nanoparticles modified with glycol with a longer chain (polyethylene glycol) possessed a higher *r*_1_ value than those modified with glycol with a shorter chain (diethylene glycol or triethylene glycol).^26^ Other studies evidenced that coating polyacrylic acid (PAA) on GONs revealed higher *T*_1_ relaxivity. This result was attributed to the hydrogen bond between carboxyl groups, leading to both the factor *q* and the speed up of water exchange (1/*τ*_m_).^17,27^ Surface coating also impacts the water diffusion. Any factors preventing the affinity between hydrated protons and the nanoparticles could have negative influence on the relaxivity because of the reduced *q* number. For example, modifying nanoparticles with double layers of amphiphilic molecules led to impaired contrast as the hydrophobic end prevents the approaching of water protons.^24,28^

Although the mechanism of the contrast enhancement has been substantially understood, practically, significant variation of *r*_1_ of the GONs with a similar structure and surface chemistry raises the question on the influences from parameters. For example, for PAA coated Gd_2_O_3_ nanoplates with a core diameter of 2 nm, the longitudinal relaxivity showed huge variation ranging from 13.28 to 70.20 mM^−1^ s^−1^ at 1.5 T,^17,24,29^ while the *r*_1_ value of GONs coated by polyethylene glycol (PEG) mentioned in the three reports also varied from 5.75 to 29.00 mM^−1^ s^−1^ at 3 T.^26,30^ The remarkable difference of *r*_1_ implied that attributing a high *r*_1_ value only to the above mentioned parameters might be limited.

Herein, we synthesized a series of GONs with adjusted microstructure by modifying a thermal decomposition method.^31^ These nanoparticles were then modified with citric acid (CA), PAA, (3-aminopropyl)triethoxysilane (APTS) and polyacrylamide (PAM), respectively, to evaluate the influence of surface modification on MRI *T*_1_ contrast. Combined with *T*_1_ relaxivity measured under various pH conditions, we integrated the already-known factors for *r*_1_ of GONs into a framework of the isoelectric point. In addition, we unexpectedly observed that different degrees of crystallinity of GONs with uniform size and same modification lead to the distinctive MRI *T*_1_ contrast. Thus, our work may shed light on the design of nanoparticulate *T*_1_ contrast agents.

## Experimental

### Materials

Oleic acid (OA, >90%), 1-octadecene (ODE, >90%), Arg-Gly-Asp (RGD, 97%), polyacrylamide (PAM, *M*_n_ = 40 000), (3-aminopropyl)triethoxysilane (APTS, 99%), sodium hydroxide (NaOH, 97%), and hydrochloric acid (HCl, 37%) were purchased from Sigma-Aldrich. Cyclohexane (99.5%), *N*-hexane (97%), ethanol (99.7%), and diethylene glycol (DEG, 98%) were purchased from General-Reagent. Sodium oleate (NaOL, 98%), gadolinium chloride hexahydrate (GdCl_3_·6H_2_O, 99.99%), and citric acid (CA, 99.5%) were purchased from Macklin. *N*,*N*-Dimethylformamide (DMF, 99.8%) and oleylamine (OM, 80–90%) were purchased from Aladdin. Poly(acrylic acid) (PAA5000, *M*_w_ = 5000, 50 wt%) and poly(acrylic acid) (PAA2000, *M*_w_ = 2000, 63 wt%) were purchased from Acros Organics.

### Synthesis of GONs

#### Synthesis of the Gd-oleate precursor

GONs were synthesized *via* modifying the thermolysis of metal-oleate complexes.^31^ Gd chloride hexahydrate (160 mmol) and NaOL (480 mmol) were added to a mixture solvent composed of ethanol (640 mL), distilled water (480 mL) and hexane (1120 mL) with magnetic stirring at 70 °C for four hours. Then 240 mL distilled water was used to extract the upper organic layer containing the Gd–oleate complex three times in a separator funnel. After the process of extraction, a yellow viscous solution was obtained. Finally, hexane was evaporated at 35 °C, resulting in the Gd-oleate precursor in a waxy solid form.

#### Synthesis of GONs

The precursor and a mixed solvent of OA, OM, and ODE were added in a three-neck round-bottom flask and heated to 100 °C under vacuum for 1 h to remove water. After bubbling in a nitrogen atmosphere for 10 min, the reaction solution was then quickly heated to the reaction temperature (310 °C/320 °C) for a period (1 h or noted in the text) under protection of bubbling nitrogen, resulting in the formation of GONs. The resultant solution containing the GONs was then cooled to 70 °C. An excess amount (∼80 mL) of ethanol was added into the solution to precipitate the GONs. Then as-precipitated nanocrystals were washed with cyclohexane and ethanol three times, collected by centrifugation and dried in air at 35 °C overnight. In our experiments, the reaction temperature, the reaction time, the amount of precursor, and the composition of OA, OM and ODE were varied, as listed in Table 1.

**Table 1:** Synthesis conditions and the resultant yield and size of a part of GONs

Sample	OA (mL)	OM (mL)	ODE (mL)	Temperature (°C)	Time (min)	Yield (%)	Size (nm)
GON5-a	12	36	32	310	60	38.4	5 ± 1.5
GON5-b	24	36	20	310	60	42.1	5 ± 1.0
GON5-c	12	66	2	320	60	46.8	5 ± 0.8
GON9-a	12	36	32	320	60	66.0	9 ± 0.7
GON9-b^a^	36	54	30	320	40	98.5	9 ± 0.4
GON17-a	12	54	14	320	60	72.6	17 ± 2.6
GON17-b^b^	12	54	14	320	60	91.7	17 ± 5.3

a Prepared with 1.2 g Gd-oleate precursor.

b Synthesized by directly decomposing the mixture of NaOL and GdCl_3_. All other samples were prepared with 0.8 g Gd-oleate precursor. The yields were the ratio between Gd in resultant GONs (measured by ICP) and that in the precursor (0.7 mmol).

### Surface modification

The as-synthesized nanoparticles that are capped with oleate ligands were surface modified by using general ligand exchange procedures and grafting reactions.

#### Synthesis of GON–CA^32^

CA coated GON was made by exchange of surface oleic acid with citric acid. GON (27 mg) was stirred with citric acid (0.2 M, 6 mL) for 16 h at room temperature. After adjusting the pH value to 10 with a dilute solution of NaOH (0.1 M), the resulting solution was dialyzed (*M*_w_ cut-off 3.5 kDa) in pure water for purification.

#### Synthesis of GON–PAA(2000/5000)^32,33^

PAA2000 (600 μL) or PAA5000 (766 μL) and 12 mL of diethylene glycol were heated to 110 °C under vacuum for 30 min. A cyclohexane solution (8 mL) containing GON (1000 mg) was slowly injected into the hot solution, while keeping the solution temperature at 110 °C under a nitrogen atmosphere, followed by rapid heating to 240 °C and keeping at this temperature for 30 min. After the solution was cooled to room temperature, 1 mL of dilute solution of HCl (0.10 M) was added to precipitate the GON–PAA. After washing with deionized (DI) water three times, the precipitate was collected by centrifugation, and then neutralized with a dilute solution of sodium hydroxide (0.05 M).

#### Synthesis of GON–PAA–RGD^34^

A DMF solution (30 mL) containing GON–PAA (90.0 mg) and RGD (12.2 mg) was stirred at room temperature for 24 h to graft the RDG to the surface of GON–PAA. The resultant solution of GON–PAA–RGD was then rinsed by dialysis (*M*_w_ cut-off 7 kDa) in pure water for purification.

#### Synthesis of GON–APTS^35,36^

GON (10 mg) was mixed with ethanol solution (20 mL) and APTS (500 μL) under stirring for 48 h at room temperature. The GON–APTS was then precipitated with ethanol, followed by rinsing with pure water three times.

#### Synthesis of GON–PAM^37^

10 mL weakly alkaline PAM aqueous solution (5 mg mL^−1^, pH 8) was stirred at room temperature for 30 min. 10 mL cyclohexane solution containing 10 mg GON and 5 mL DMF was sequentially injected into the PAM solution under stirring for 4 h. The resulting reaction liquid was allowed to stand, and the lower layer liquid was retained. After washing twice with ethanol, the precipitate was collected by centrifugation, and then dispersed in an aqueous solution.

### Characterization

The size and morphology of the nanocrystals were observed with a TALOS L120C transmission electron microscope (TEM). The high-resolution images were recorded on a TALOS F200X high resolution transmission electron microscope (HRTEM), with which the selected area electron diffraction (SAED) patterns were also obtained. Samples for TEM were prepared by depositing a drop of GONs dissolved in hexamethylene or deionized water onto carbon-coated copper grids and evaporating the solvent immediately. The concentration of Gd in the solution sample was determined *via* a Thermo Scientific™ iCAP™ 7600 ICP-OES inductive coupled plasma emission spectrometer (ICP). The X-ray diffraction patterns were recorded with a D8 Advance Da Vinci X-ray diffractometer (Bruker Co.). Zeta potential measurements under different pH were performed with a NanoBrook Omni to determine the isoelectric point. The relaxivities and *T*_1_-weighted MR images were obtained using a MesoMR23-060H-I (0.5 T, Shanghai Electronic Technology Co.).

## Results and discussion

### Microstructural adjustment of GONs

**Fig. 1:**
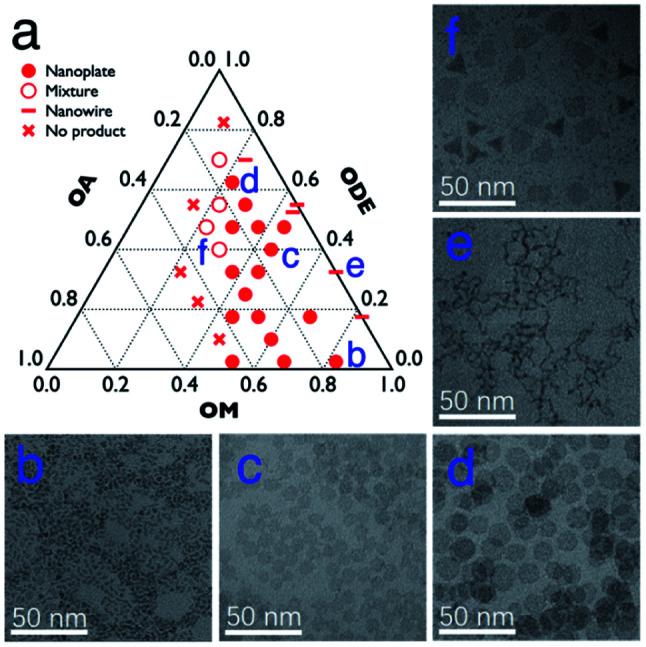
(a) The outcomes with 800 mg Gd-oleate precursor at 320 °C for 1 h with different volume ratios of OA, OM and ODE; and (b–f) TEM images of the corresponding samples labelled in a.

In general, the Gd–oleate complex was prepared as the precursor of GONs by the reaction between GdCl_3_ and sodium oleate. Then the precursor was decomposed in a mixture of OA, OM, and ODE, among which OA and OM served as the ligands, while ODE acted as the solvent. We at first evaluated the influence of the ratio of OA/OM/ODE. A ternary phase diagram (Fig. 1a) schematically showed the outcome of the protocol, and the typical morphologies of the resultant nanoparticles are presented in Fig. 1b–f. All the syntheses in the figure were conducted using 800 mg precursor ([Gd^3+^] = 0.7 mmol) by decomposing it at 320 °C for 1 hour. In most cases (marked as round solid points in Fig. 1a, and presented as Fig. 1b–d), the GONs were in a nanoplate shape that the flat faces were near round. Meanwhile, other shapes, such as nanowires (Fig. 1e), irregular-shaped nanoplates, nanotriangles, and their mixture (Fig. 1f), were obtained with other ratios of OA/OM/ODE. Additionally, GON was not produced under some conditions, especially when OM : OA < 1 : 1 (the left half of the ternary phase diagram). Among those conditions forming nanoplates, the influence of OM and OA on the diameter of the nanoplates was evaluated, as shown in Fig. S1 and S2, ESI.† With the elevation of OM percentage, the particle size decreased gradually, whereas the increased OA in the reaction solution in general leads to a larger size of nanoparticles. It has been reported that OM possesses the capability of promoting the nucleation process during a thermo-decomposition process.^38^ Thus, the increase of OM resulted in more number of nuclei, which contributes to the decreased size of resultant nanoparticles. In contrast, OA can adhere to the precursor molecules or the surface of the formed GON nuclei during the growth stage.^39^ This attachment prevented nanoparticles from rapid agglomeration and fast growing. This attachment was also suggested by the cases that the solvent almost did not contain OA. In these cases (en dash in Fig. 1), nanowires were obtained, indicating that the small nuclei would conjugate one by one without the protection of OA.^16^ In contrast, when the amount of OA in the reaction solution increases, the OA conjugating to the active monomers led to the difficulty of nucleation, resulting in larger nanoplates. Additionally, when the dose of OA increased to a certain range (in our case, more than that of OM), the nucleation was inhibited, resulting in no products.

**Fig. 2:**
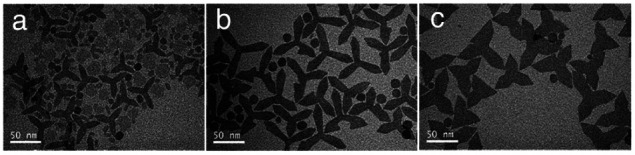
Typical TEM images of decomposing 8 g Gd-oleate in a mixture of 24 mL OA, 36 mL OM, and 20 mL ODE at 320 °C for (a) 1 h, (b) 2 h, and (c) 3 h.

Apart from the ratio of OA/OM/ODE, other parameters could also be utilized to adjust the microstructure. For example, all of the remaining reaction conditions were unchanged, and shortening the reaction time reduced the diameter of GONs0020 (shown in Fig. S3, ESI†). Meanwhile, doubling or tripling the concentration of the precursor also led to the smaller size of nanoplates. Further, we unexpectedly observed that if the feeding dose increased to 10-fold higher (to 8 g Gd-oleate), a mixture of round nanoplates and tripodal GONs was produced (Fig. 2a). In this case, when we elongated the reaction time from 1 h to 3 h, most of the nanoplates disappeared, whereas the tripodal GONs grew in both width and length of each arm (Fig. 2). C. B. Murray *et al.* reported^21^ that tripodal GONs could be synthesized by decomposing gadolinium acetate in OA/OM/ODE with the help of lithium hydroxide. They highlighted the crucial role of Li^+^ in the morphology control as the substitution by NaOH didn't lead to the same shape. In contrast, we showed that tripodal GONs can be synthesized without adding any other metal ions. Besides, we noticed that if the raw materials, sodium oleate and gadolinium chloride, were directly mixed in the solution of OA, OM, and ODE, GONs were still obtained without the preparation of the Gd–oleate complex (Fig. S4, ESI†).

**Fig. 3:**
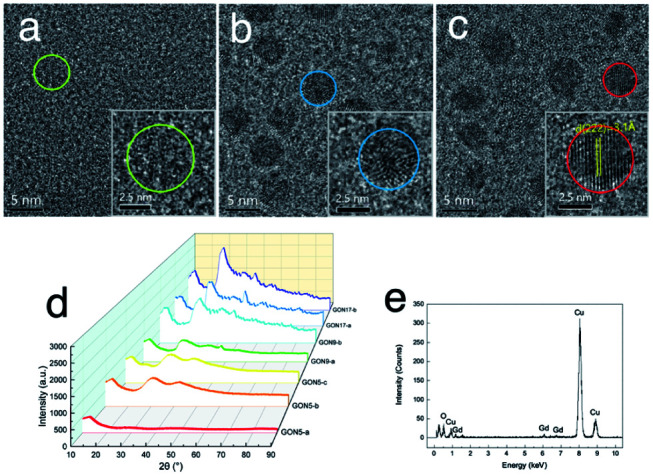
Characterization of GONs, (a–c) HRTEM images of GON5-a, GON5-b and GON5-c. The insets presented one enlarged nanoplate of the corresponding samples. Their boundary was circled. (d) XRD pattern of GONs. (e) EDS result of the sample GON5-a.

Aiming at studying the MRI contrast of GONs, we focused on three distinct sizes of monodisperse nanoplates synthesized under different conditions (listed in Table 1). The TEM images of a series of as-obtained nanoparticles are shown in Fig. S4, ESI,† presenting a uniform diameter of 5 nm (Fig. 3a–c), 9 nm (Fig. S2b† for GON9-a and Fig. S3b† for GON9-b), and 17 nm (Fig. S4, ESI†), respectively. As illustrated in Fig. 3c, GON5-c showed a regular crystalline lattice with an interplanar distance of *d* ≈ 3.1 Å, which matched to the (222) plane of the cubic Gd_2_O_3_ phase. Contrary to the ordered crystal of GON5-c, the HRTEM images of GON5-b (Fig. 3b) and GON5-a (Fig. 3a) illustrated a crystal with defects, and an amorphous particle, respectively. The different crystallinity can be further evidenced by their X-ray diffraction (XRD) patterns. As shown in Fig. 3d, XRD patterns demonstrated that cubic Gd_2_O_3_ nanoparticles (JCPDS-86-2477) were obtained, and the broadening of diffraction peaks should be ascribed to the nano-scale size. Notably, although with the same size of 5 nm, the more disordered GONs that were observed by HRTEM possessed wider XRD peaks. Similar phenomena were recorded between the XRD patterns of samples GON9-a and GON9-b, and between those of GON17-a and GON17-b. These results further proved the variation of the crystallinity among the GONs with the same size. Besides, the energy dispersive X-ray spectrum (EDS) in Fig. 3e showed that the GONs were composed of Gd and O elements.

### MRI contrast affected by the isoelectric point

**Fig. 4:**
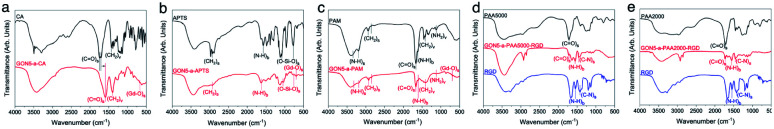
FT-IR absorption spectra of GON5-a, (a) –CA, (b) –APTS, (c) –PAM, (d) –PAA5000–RGD, and (e) –PAA2000–RGD along with their respective coatings.

Five hydrophilic molecules, namely CA, PAA2000, PAA5000, PAM, and APTS, were coated on the GONs, respectively, to evaluate the influence of acidity on the MRI contrast. All the modified nanoparticles showed excellent water solubility, indicating successful modification. Fig. 4a–c present the FT-IR spectra of GONs–CA, –APTS and –PAM, respectively, with the corresponding modified molecules as the references. The characteristic peak observed near 690 cm^−1^ for all the samples corresponded to the stretching vibration of Gd–O in Gd_2_O_3_.^40^ The C=O stretching vibrations at 1743 and 1708 cm^−1^ in the FT-IR absorption spectrum of the GONs–CA were red-shifted from 1592 cm^−1^ of the pure CA due to the electrostatic bonds between the COO^−^ groups of each CA and surface Gd^3+^ ions of each GON. The peaks at 1076 and 794 cm^−1^ of GONs–APTS could be attributed to the antisymmetric and symmetric stretching vibrations of the Si–O–Si bond of the APTS group, respectively. The antisymmetric and symmetric stretching vibrations of the N–H bond at 3350 and 3185 cm^−1^, as well as the C=O stretching vibration at 1664 cm^−1^ in GON–PAM were the typical signals which supported the successful coatings. Fig. 4d and e present the FT-IR absorption spectra of GON–PAA2000–RGD and GON–PAA5000–RGD, respectively, compared with those of the neat PAA and RGD molecules. These two kinds of coated GONs exhibited a peak near 1720 cm^−1^ due to the C=O stretching vibration in PAA, and other two peaks at 1554 and 1456 cm^−1^ given by the N–H bending and C–N stretching in RGD, confirming the surface coating of the GON with both PAA and RGD.

**Fig. 5:**
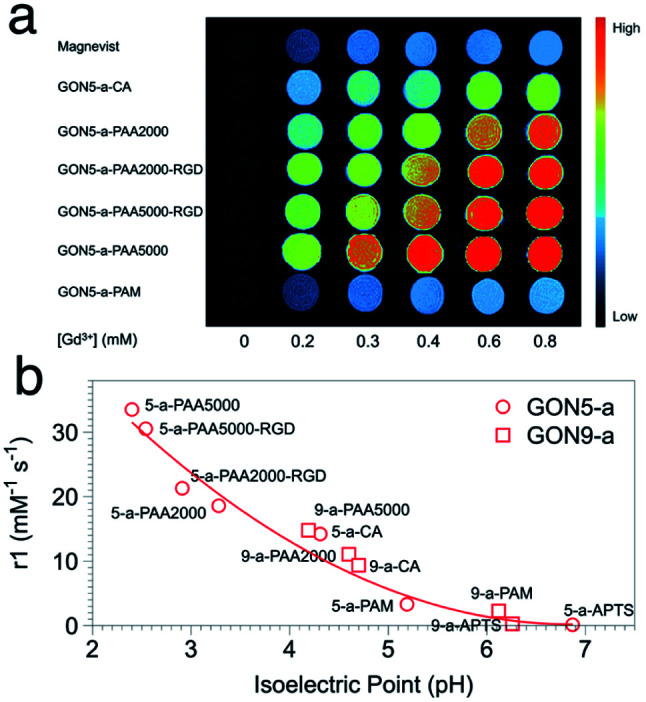
(a) The MR images of GON5-a modified with different ligands, with Gd–DTPA (Magnevist) as a control. (b) *r*_1_ value of different coated GONs as a function of their isoelectric points.

In the evaluation of *T*_1_ relaxation, we firstly recorded the *r*_1_ value of clinically used Gd–DTPA as a reference, which was 4.46 mM^−1^ s^−1^. Those of GON5-a and GON9-a with different modifications varied from 33.52 to 0.14 mM^−1^ s^−1^ and 14.79 to 0.29 mM^−1^ s^−1^, respectively (as listed in Table S1, ESI†). For example, among the modified samples GON5-a, GON5-a–PAA5000 showed the highest *r*_1_ value of 33.52 mM^−1^ s^−1^ in neutral solvent. With a shorter chain length of ligand, GON5-a–PAA2000 retained a relatively low *r*_1_ value of 26.35 mM^−1^ s^−1^. This discrepancy was consistent with the reported case of PEG modified GONs. We also chose an oligopeptide, Arg-Gly-Asp (RGD), a ligand that can target the RGD receptor overexpressed tumors to graft to the PAA coated GON5-a. The *T*_1_ relaxivity of GON5-a–PAA2000–RGD increased to 27.20 mM^−1^ s^−1^, while that of GON5-a–PAA5000–RGD slightly decreased to 30.54 mM^−1^ s^−1^ under 0.5 T. The high relaxivity was also confirmed by the phantom images presented in Fig. 5a. At the same concentration, *T*_1_-weighted MR images of GON5-a–PAA5000–RGD showed much brighter contrast than commercially available Gd–DTPA molecules. These results were, to some extent, consistent with those reported results. For instance, the relaxivity went higher as the chain length of the ligand increased.^26^ Meanwhile, GONs coated with PAA manifested outstanding *T*_1_ relaxivity, which previously was ascribed to the hydrogen bonds between PAA and water molecules.^27,29,41,42^ However, GON5-a–PAM, in which hydrogen bonds between amine groups and water molecules also exist, possessed an undesirable *r*_1_ of only 3.31 mM^−1^ s^−1^. In the meantime, for 9 nm particles, the CA modified GON has a slightly higher relaxivity than the PAA2000 modified one, which was unexpected.^43^

Nonetheless, our results appeared to be associated with the acidity and alkalinity on the surface of the nanoparticles. We thus determined the isoelectric points (pI) of all the modified GONs and correlated them with the *r*_1_ values. The results showed that the *r*_1_ relaxivity decreases with the increase of their isoelectric point (Fig. 5b). For example, GON5-a–PAA5000 with the highest relaxivity had the lowest isoelectric point (pI = 2.40); in contrast, the sample modified with APTS with a minimum *r*_1_ value has the highest isoelectric point (pI = 6.26). Since the isoelectric point is the environmental pH value when there is no charge on the surface of nanoparticles, it describes the ability of the surface to dissociate hydrogen protons.^44^ A smaller pI means that in a neutral solution, more H^+^ could be dissociated from the surface of the nanoparticles (*q* increases) and affected by the paramagnetic Gd^3+^ strongly (*τ*_m_ and *τ*_R_ increase), thus contributing to the imaging contrast. It should be mentioned that these dissociated protons are in addition to those existing in the water. Contrarily, when the isoelectric point is close to 7 (-PAM or -APTS), the additional protons can hardly be dissociated. Thus, the *r*_1_ values were close to that of Gd–DTPA. The influences of already-known factors with surface modification, including hydrogen bonding, hydration radius, hydrophilicity, *etc.*, may be explained by the isoelectric points, as all these factors dictate the change of pI.^45^

It should be noted that the impact of isoelectric points may also cover that of the size of GONs. For the nanoparticles with certain composition, phase, and surface modification, a smaller size or larger surface-to-volume ratio leads to that more H^+^ protons in the environment are needed to inhibit the dissociation of H^+^ on the surface of nanocrystals, which means that smaller sized nanoparticles have a smaller value of pI.^46^ Hence, when we put the points of GON9-a into Fig. 5b, they also fit well with the trend of GON5-a. Still, the lower pI of GON results in the better capability of generating additional H^+^ in neutral solvent, resulting in a higher relaxation rate. Therefore, the isoelectric point could be a direct indicator of the relaxivity, regardless of the size or surface modification of the nanoparticles.

Thus, the imageable H^+^ protons could be categorized into two parts: those exist in water and those dissociated from the modified surface of nanoparticles. We proposed that the latter part played a pivotal role in MRI *T*_1_ imaging. If the modified surface of a nanoparticle dissociates more H^+^, the nanoparticle has a better imaging contrast. Additionally, because nanoparticles with “inappropriate” modification present comparable *r*_1_ with Gd–DTPA, the enhancement of the contrast might have predominantly originated from the latter type.

**Fig. 6:**
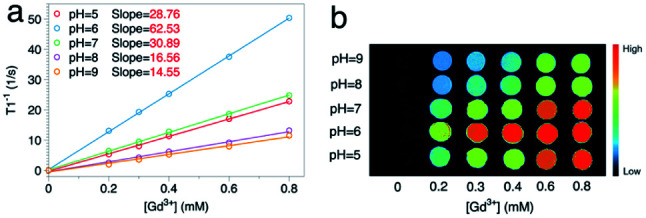
(a) 1/*T*_1_ and (b) the MRI phantom images of the sample GON5-a–PAA5000 at different pH values (25 °C, 0.5 T).

To further verify this, pH values of the solvent were adjusted by adding diluted hydrochloric acid or sodium hydroxide solution. The influence of the environmental pH should be double fold: on one hand, lowering pH increases the number of protons in the solvent (the former kind); on the other hand, it makes the ionization of modified molecules harder (decreasing the latter type). Taking GON5-a–PAA5000 as an example (Fig. 6), when the pH was lowered from 9 to 6, the longitudinal relaxivity increased to their maximum, 62.53 mM^−1^ s^−1^ (1/*T*_2_*vs.* the concentration is shown in Fig. S5†). This result was close to the reported highest *r*_1_ value^17^ and indicated that the concentration of H^+^ in the environment made contributions to the contrast. However, when we further increase the concentration of H^+^, the *r*_1_ value decreases to 28.76 mM^−1^ s^−1^ at pH = 5. This phenomenon can only be explained by the shift of chemical balance of the ionization, which reduced the number of dissociated H^+^ from the nanoparticle surface. Furthermore, the dependence of the contrast of GONs on environmental pH possesses the potential to be applied in active imaging of tumors because of the acidity of the tumor microenvironment.

### Crystallinity: the hidden factor

We also measured *r*_1_ and *r*_2_ of the samples with various crystallinities yet the same size and citric acid modification. Unexpectedly, with the decreased crystallinity, the *r*_1_ value increased from 9.41 to 14.21 mM^−1^ s^−1^, and the *r*_2_/*r*_1_ ratio varied from 1.05 to 1.25 for GON5-c–CA and GON5-a–CA, respectively. For those nanoparticles of 9 nm and 17 nm, still the GONs with a lower degree of crystallinity showed a higher relaxation rate (Table S1, ESI†). The relaxivities of GONs turned out to be dramatically different despite the same size and modification. The more defects the GONs possessed, the higher relaxivities the GONs presented.

**Fig. 7:**
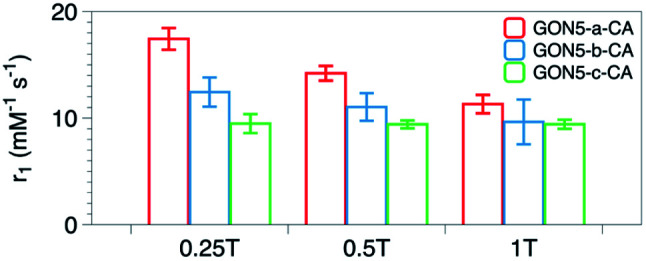
*r*_1_ values of the samples GON5-a–CA, GON5-b–CA, and GON5-c–CA under the applied field of 0.25 T, 0.5 T and 1 T, respectively.

To explain this unusual phenomenon, we proposed that the electronic relaxation (*τ*_s_) might be the main contribution. The influence of *τ*_s_ depends mainly on the decay of the electron spin magnetization.^47^*τ*_s_ is the dominant correlation time at very low fields (<0.1 T) and is inversely related to *r*_1_. For high spin ions like Gd^3+^, the electronic relaxation largely depends on the interelectronic interactions in paramagnetic compounds with two or more unpaired electrons.^48^ In the studies of classical Gd chelates, generally one Gd^3+^ existed in one molecule. Thus, the decrease of *τ*_s_ only happens when the distance between Gd ions in different molecules is significantly shortened. This is hard to occur in a solution state. Therefore, its contribution to the relaxivity of Gd chelates is negligible and *τ*_s_ was usually not considered in the development of small-molecule contrast agents. However, Gd ions within one certain nanoparticle are close, which may shorten *τ*_s_ into an influential range. Based on this, owing to that the defects in the nanocrystals could shorten the distance between metal ions,^49^ further decrease of *τ*_s_ could be expected. Owing to that *τ*_s_ proportionally increases with the square of increasing applied field, its contribution could manifest on relaxivity more significantly at low fields than at high fields. Thus, we detected *r*_1_ of the nanoparticles under an applied magnetic field of 0.25 T, 0.5 T and 1 T respectively. As shown in Fig. 7b, the difference among samples decreases with the increase of applied field, which confirms that *τ*_s_, or the distance between Gd ion inter-nanoparticles, contributes to the MRI contrast.

Although shortening of *τ*_s_ by the intramolecular electronic relaxation between Gd^3+^ ions in classical chelate molecules has been observed in their solid form,^50,51^ few studies considered its contribution in nanoparticles. An only example was reported by Gao *et al.*^52^ who reported a method to substitute the Fe^2+^ ions in magnetite nanoparticles by Mn^2+^. This substitution resulted in a remarkably higher *r*_1_ than their parent iron oxide nanoparticles. They ascribed their results to the 5 unpaired electrons of Mn^2+^ and the consequent change of *τ*_s_. Combined with their work, utilizing electronic relaxation may possess promising potential in developing nanoparticulate MRI contrast agents.

## Conclusions

We synthesized a series of Gd_2_O_3_ nanoparticles with adjustable size, shape, and crystallinity. By modifying the molecules with different acidities, we showed that the longitudinal relaxivity of the nanoparticles was dependent on the isoelectric points. Besides surface modification, the influence of nanoparticles size was also integrated into the framework of isoelectric points. Furthermore, we showed that the crystallinity of the nanoparticles also has an impact on the relaxivity. Our results presented that the difference in *r*_1_ among samples with different crystallinities was more obvious in the lower applied field, and thus, we ascribed the difference to the contribution of electronic relaxation. Our work clarified how the parameters affect the longitudinal relaxivity of Gd_2_O_3_ nanoparticles, which may shed light on developing high-performance nanoparticulate *T*_1_ contrast agents.

## Conflicts of interest

There are no conflicts to declare.

## Supplementary Material

NA-004-D1NA00612F-s001
