# Supplementary material for: Revisiting the factors influencing the magnetic resonance contrast of Gd2O3 nanoparticles
Source: Nanoscale Adv. 2021 Oct 25;4(1):95–101. doi: 10.1039/d1na00612f (PMC9418219; doi:10.1039/d1na00612f)
Supplement: NA-004-D1NA00612F-s001 [file NA-004-D1NA00612F-s001.pdf]

Electronic Supplementary Material (ESI) for Nanoscale Advances.
This journal is © The Royal Society of Chemistry 2021

Electronic Supplementary Information

Revisiting the factors influencing magnetic resonance contrast of Gd_2O_3 nanoparticles

Yanyue Liu,[a] Yingfan Dai,[a] Haifeng Li,[a] Dida Duosiken,[a] Na Tang,[a] Kang Sun,[a] and Ke Tao*[a]

a. State Key Lab of Metal Matrix Composites, School of Materials Science and Engineering, Shanghai Jiao Tong University, Shanghai 200240, P. R. China.

*Correponding Author: ktao@sjtu.edu.cn

Figure S1.

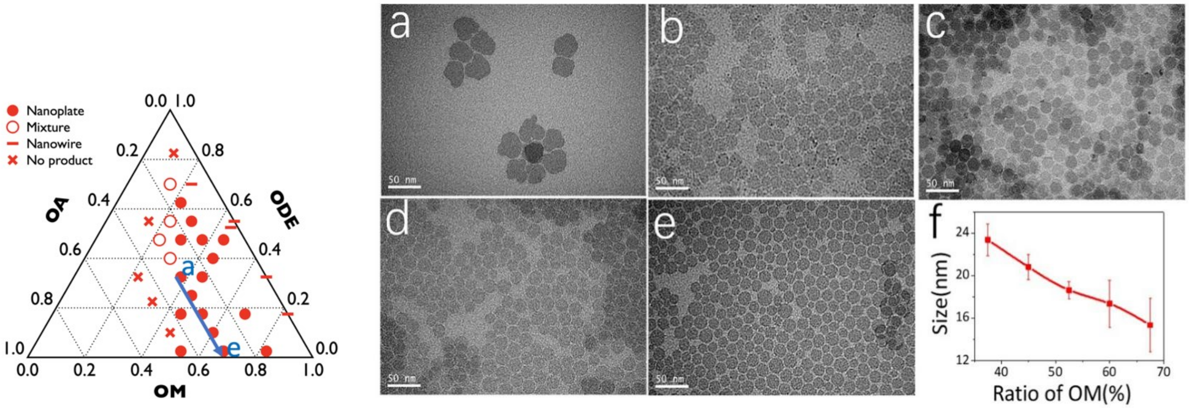

Figure S1. The influence of OM percentage on the size of nanoplate, images a-e corresponded to the five dots on blue arrow in left panel (OM = 37.5%, 45%, 52.5%, 60% and 67.5%), respectively, and OA was fixed as 30%. A plot (f) presented their size evolution.

Figure S2.

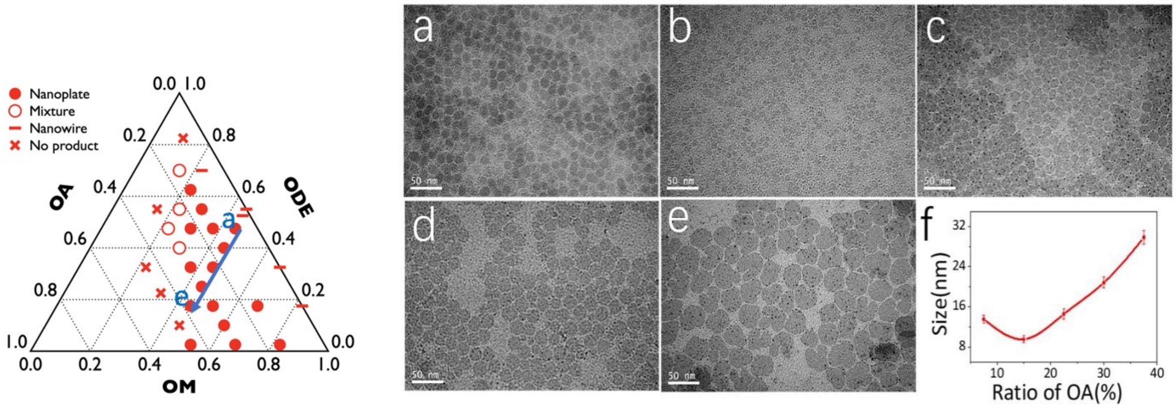

Figure S2. The influence of OA percentage on the size of nanoplate, images a-e corresponded to the five dots on blue arrow in left panel (OA = 7.5%, 15%, 22.5%, 30% and 37.5%), respectively, and OM was fixed as 45%. Notably, Fig. S2d=Fig. S1b. A plot (f) presented their size evolution.

Figure S3.

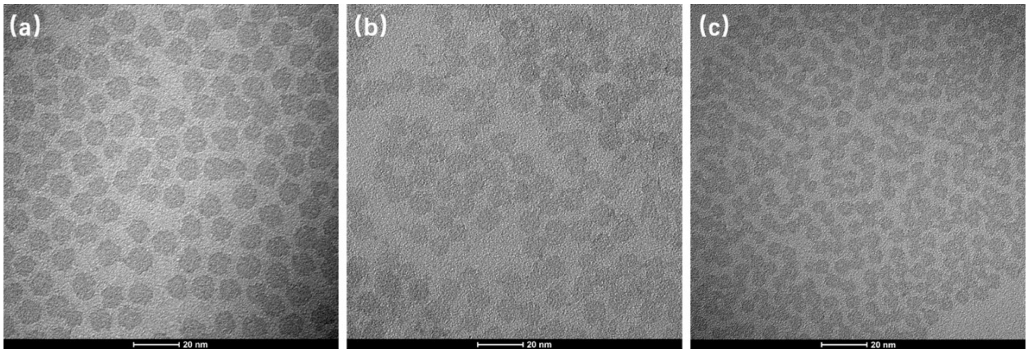

Figure S3. TEM images of decomposing a) 800mg, b)1200mg and c)1600mg Gd-oleate precursor in a mixture of 6mL OA, 36mL OM and 38mL ODE under 320°C for 1h.

Figure S4.

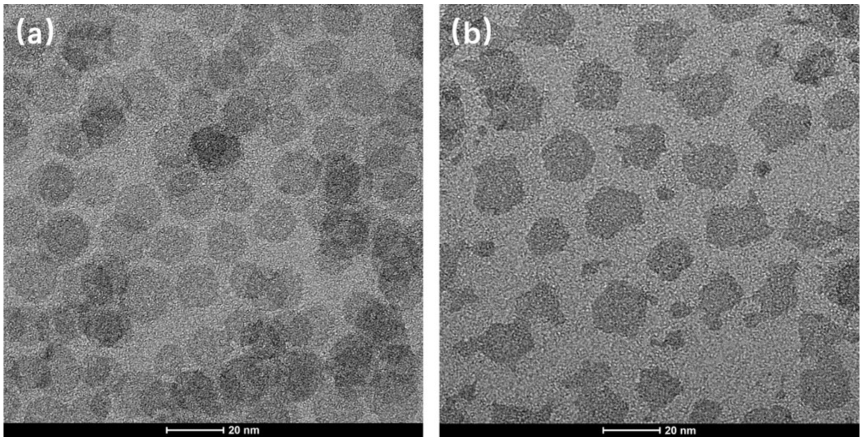

Figure S4. TEM images of sample a) GON17-a and b) GON17-b, the latter one was prepared by directly heating the mixture NaOL and $GdCl_3$ in OA/OM/ODE without preparing the precursor.

Figure S5

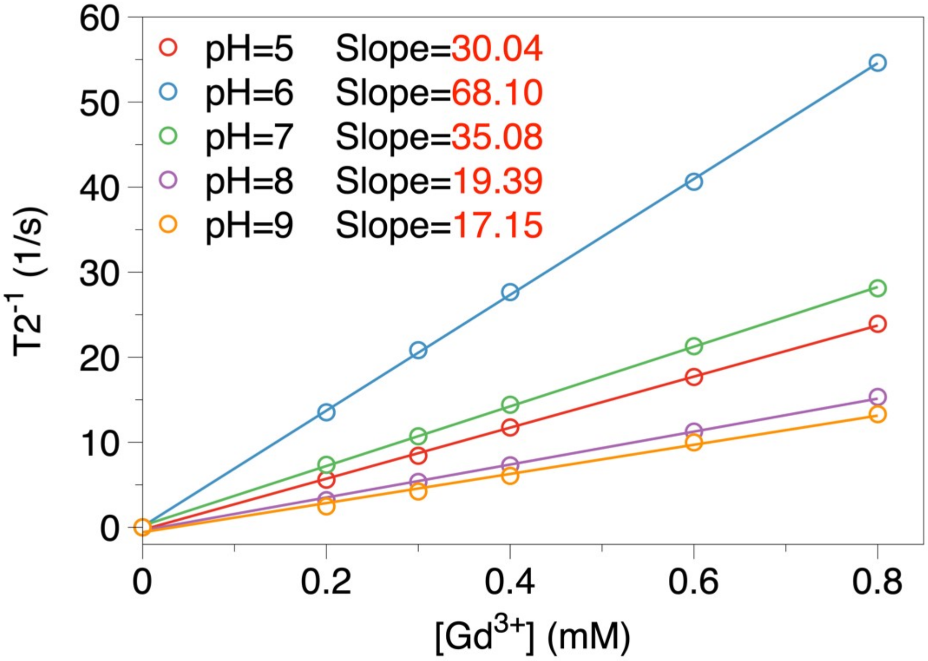

Figure S5. 1/T2 of the sample GON5-a-PAA5000 in the environment of different pH.

Table S1. Relaxivities r1, r2 and r1/r2 ratios of the GONs from the slopes of the plot of 1/T and concentration of Gd^{3+} at 0.5 T.

Sample nomenclature	r1 ($mM^{-1} \cdot s^{-1}$)	r2 ($mM^{-1} \cdot s^{-1}$)	r2 / r1
Gd-DTPA	4.458	5.037	1.13
GON 5-a-CA	14.207	15.801	1.11
GON 5-a-PAA2000	26.350	31.795	1.21
GON 5-a-PAA2000-RGD	27.199	32.037	1.18
GON 5-a-PAA5000-RGD	30.539	35.081	1.15
GON 5-a-PAA5000	33.519	37.493	1.12
GON 5-a- PAM	3.311	6.193	1.87
GON 5-a-APTS	0.140	0.943	6.74
GON 5-b-CA	10.045	12.518	1.25
GON 5-c-CA	9.414	9.866	1.05
GON 9-a-CA	11.061	12.449	1.13
GON 9-a-PAA2000	9.359	11.383	1.22
GON 9-a-PAA5000	14.793	17.792	1.20
GON 9-a-PAM	2.295	5.402	2.35
GON 9-a-APTS	0.292	1.518	5.20
GON 9-b-CA	8.632	10.212	1.18
GON 17-a-CA	9.245	10.986	1.19
GON 17-b-CA	7.919	9.083	1.15
